# A Novel Simple Assay System to Quantify the Percent HCV-RNA Levels of NS5A Y93H Mutant Strains and Y93 Wild-Type Strains Relative to the Total HCV-RNA Levels to Determine the Indication for Antiviral Therapy with NS5A Inhibitors

**DOI:** 10.1371/journal.pone.0112647

**Published:** 2014-11-14

**Authors:** Yoshihito Uchida, Jun-ichi Kouyama, Kayoko Naiki, Satoshi Mochida

**Affiliations:** Department of Gastroenterology & Hepatology, Saitama Medical University, Saitama, Japan; Scripps Research Institute, United States of America

## Abstract

**Aim:**

Oral treatment with asunaprevir and daclatasvir has been reported to yield a SVR ratio of 80% in patients with genotype 1b HCV infection, however, treatment failure has been reported, especially in patients with HCV strains showing the NS5A-Y93H mutation at baseline. An assay system to detect such strains was established to facilitate selection of appropriate candidates for this antiviral therapy.

**Methods:**

Primer sets and 2 types of cycling probe mixtures were designed, and real-time PCR was performed with HCV-RNA purified from 332 patients with genotype 1b HCV infection, and the results were compared with those obtained by direct sequencing.

**Results:**

Both the wild-type and mutant strains were quantified, with a threshold of 4.0 Log copies/mL, in 295 of the 332 patients (88.9%), and the percentage of the mutant strains relative to the total HCV-RNA level in the serum was calculated. The percentage was 0% in 237 patients (80.3%) and 100% in 23 patients (7.8%), identical to the results of direct sequencing. Both wild-type and mutant strains were detected in the remaining 35 patients (11.9%), at levels between 1% and 99%, despite the mutant strains having been undetectable by direct sequencing in 11 patients with percentages of these strains of less than 25%.

**Conclusion:**

A novel assay system to quantify the percent RNA of Y93H mutant strains relative to the total HCV-RNA level was established. This system may be useful to determine the indication for treatment with NA5A inhibitors in patients with HCV.

## Introduction

Hepatitis C virus (HCV) infection is a global health problem. An estimated 185 million people worldwide suffer from persistent HCV infection [1]. These patients are at a serious risk of developing the complications of hepatic cirrhosis and hepatocellular carcinoma (HCC) [2], and globally, approximately 350,000 deaths per year are attributed to cirrhosis and HCC caused by HCV infection [3]. In Japan, more than 30,000 people die as a result of HCC each year [4], and in 70% of these cases, the etiology has been shown to be HCV infection [5].

Combined pegylated interferon (Peg-IFN) plus ribavirin therapy was the standard therapy for patients with chronic hepatitis caused by HCV until 2011. Sustained viral response (SVR) was obtained in about 70% of patients with genotypes 1a and 1b infections when response-guided therapy (RGT) was adopted, that is, the treatment duration was prolonged from 48 to 72 weeks in patients in whom undetectable serum HCV-RNA failed to be achieved at 12 weeks (complete early viral response: cEVR) [6], [7]. The antiviral efficacy of combined Peg-IFN plus ribavirin therapy in patients with chronic hepatitis C was improved by the addition of telaprevir [8]–[10] and boceprevir [11], both first-generation NS3/4A protease inhibitors. In Japan, telaprevir was approved in November 2011 as an antiviral agent for the treatment of patients with chronic hepatitis caused by genotype 1 HCV infection, both those without a previous history of combined Peg-IFN plus ribavirin therapy [12] and those who developed relapse or showed no response to previous combined Peg-IFN plus ribavirin therapy [13]. SVR was obtained in about 80% of the patients after the triple therapy with telaprevir for 24 weeks, including in those who were over 60 years of age [14]. Such triple therapy, however, often caused various adverse effects that could potentially necessitate treatment discontinuation, such as dermatitis, hemolytic anemia, hyperuricemia, renal impairment and retinopathy [15], [16].

Both the efficacy and safety of triple therapy were expected to improve markedly following the approval of simeprevir, a second-generation NS3/4A protease inhibitor [17], which was approved in December 2013 in Japan. On the other hand, oral therapy with 2 or 3 direct-acting antiviral agents (DAAs) will come to be applied for patients with HCV in the near future, both in Japan as well as in Europe and the United States. Dual oral therapy with asunaprevir, a second-generation NS3/4A protease inhibitor, and daclatasvir, an NS5A inhibitor, for 24 weeks produced SVR ratios of about 80% in patients with genotype 1b HCV infection; responses were obtained in both non-responders to previous combined Peg-IFN plus ribavirin therapy and those who were intolerant to or ineligible for the combination therapy [18], [19]. Thus, a therapeutic strategy to select antiviral therapies with and without Peg-IFN and/ribavirin needs to be established.

Viral failure during or after the dual oral therapy with daclatasvir and asunaprevir developed mostly in patients with pre-existing Y93H mutation of the genotype Ib HCV strain, a NS5A polymorphism associated with resistance to daclatasvir [18]–[20]. In these patients, the Y93H mutant HCV strains frequently developed the L31M/V mutation in the NS5A region and L168A/V mutation in the NS3 region during the dual oral therapy, which resulted in complete acquired resistance to both daclastavir and asunaprevir [20]. The Y93H mutation was detected by direct sequencing analysis in 8.3% of genotype 1b HCV strains obtained from 362 Japanese patients without a previous history of DAA therapy [21]. Thus, knowledge about the baseline profile of NS5A polymorphism is crucial to predict the therapeutic efficacy of dual oral therapy with daclatasvir in patients with HCV infection, and a simple method for the detection and quantification of HCV strains showing the Y93H mutation needs to be established for optimized antiviral therapy, that is, should the patient receive oral therapy with DAAs alone or DAAs in combination with Peg-IFN and/or ribavirin therapy.

In the present paper, we report on a novel simple assay system to detect and quantify NS5A Y93H-mutant HCV strains by real-time PCR using cycling probes, and discuss the usefulness of this system for appropriate selection of candidates among patients with HCV infection for therapy with DAAs with or without Peg-IFN and/or ribavirin.

## Patients and Methods

### Patients and Blood Samples

The subjects were 332 patients with chronic hepatitis caused by HCV infection, consisting of 144 men and 188 women, ranging in age from 26 to 89 years old. There were 186 treatment-naïve patients, 72 with relapse and 69 non-responders after combined Peg-IFN plus ribavirin therapy, and 5 patients showing viral breakthrough during the previous therapy. None of the patients had received antiviral therapy with NS3/4A protease inhibitors, NS5A inhibitors or NS5B inhibitors. The HCV genotype, determined using the HCV GENOTYPE Primer Kit (Institute of Immunology Co., Ltd., Tokyo, Japan), was 1b in all the patients. Serum HCV-RNA levels were measured by the COBAS AmpliPrep/COBAS TaqMan HCV Test (Roche Diagnostics K.K., Tokyo, Japan). The demographic and clinical features of the patients are summarized in ***Table 1***.

**Table 1:** The Demographic and Clinical Features of Patients Subjected to Detection and Quantification of Y93 Wild and Y93H Mutant HCV Strains by Real-Time PCR Using Cycling Probe Mixtures.

	All patients (n = 332)	Quantified through real-time PCR using cycling probe mixutures (n = 295)	
		Those detected of Y93 Wild Strain (n = 237)	Those detected of Y93H Mutant Strain (n = 58)
Age	67.0±11.3^a^	66.9±11.7	67.7±10.4
Men: Women	144: 188^b^	106: 131	18: 40
Previous therapy (Naïve: Relapser: No-responder: BTH^1^)	186: 72: 69: 5^b^	130: 49: 53: 5	37: 14: 7: 0
ALT (IU/L)	41.6±32.4^a^	40.6±30.3	41.5±35.9
Albumin (g/dL)	3.9±0.5^a^	3.9±0.5	4.0±0.5
γGTP (IU/L)	40.6±55.7^a^	40.6±59.3	29.9±26.0
Creatinine (mg/dL)	0.84±0.10^a^	0.83±0.94	0.79±0.99
Total Cholesterol (mg/dL)	156.9±34.3^a^	155.8±35.4	161.0±30.1
Total Bilirubin (mg/dL)	0.8±0.5^a^	0.82±0.53	0.78±0.36
α-fetoprotein (ng/mL)	38.6±354.4^a^	49.7±419.3	8.0±11.0
HCV-RNA (Log copies/mL)	6.4±1.1^a^	6.3±1.0*	6.7±0.8
White blood cell (10^3^/mL)	4.73±1.75^a^	4.68±1.81	4.94±1.57
Hemoglobin (g/dL)	13.1±2.0^a^	13.0±2.1	13.1±1.8
Platelets (10^3^/mm^3^)	148±61^a^	148±62	153±60
HCC (positive: negative)	57: 275^b^	41: 196**	4: 54

1 BTH indicates viral breakthrough during the previous therapy.

a mean ± SD,

b Number of patients

*p<0.05 by *t*-test,

**p<0.05 by Fisher's exact test vs Those detected of Y93H Mutant Strain.

HCV-RNA was purified from the sera of patients using the QIAamp MinElute Virus Spin Kit (Qiagen K.K., Tokyo, Japan). Then, cDNA was synthesized from the obtained RNA samples using the PrimeScript RT reagent Kit (Perfect Real Time; TaKaRa Bio Inc, Seta, Japan) or cDNA Synthesis Kit (M-MLV version: TaKaRa Bio Inc.) and subjected to NS5A polymorphism analysis. Written informed consent was obtained from all the patients prior to collection of the blood samples, which was conducted with the approval of the Institutional Review Board of Saitama Medical University.

### Establishment of the Assay System for Detection of the NS5A Y93H Mutation of the HCV Strains

A set of primers, Y93_1L (nt208–226) and Y93_1R (nt376–394) primers, and 2 types of cycling probe mixtures (Ycnc and Hcnc: nt271–280), shown in ***Table 2***, were designed targeting the nucleotide sequence of the NS5A region of 295 genotype 1b HCV strains reported in Japan [21], and synthesized with 6-carboxyfluorescein (FAM) labeling for each cycling probe by TaKaRa Bio Inc. Also, 4 types of oligonucleosides (SYN-NS5A_Ycac, SYN-NS5A_Hcac, SYN-NS5A_Ycgc and SYN-NS5A_Hcgc) corresponding to the sequences between nt271 and nt281 were synthesized by TaKaRa Bio Inc (***Table 2***).

**Table 2:** Sets of Primers, Cycling Probes and Synthesized NS5A-Oligonucleosides Used for Real-Time PCR and Direct-Sequencing to Detect Y93 Wild and Y93H Mutant HCV Strains.

Name	Nucleoside Sequences (5′→3′)	Nt Position
***Primers***		
Y93-1L	5′-GGTTCCATGAGGATCGTTG-3′	208–226
Y93-1R	5′CCGTCACGTAGTGGAAATC-3′	376–394
Y93-2L	5′-GACTGACTTCAAGACCTGG-3′	48–66
Y93-2R	5′-TAACCTCCACGTACTCCTC-3′	346–364
Y93-3L	5′-AGGGATGTTTGGGACTGG-3′	16–33
***Cycling Probes for the First-Step Real-time PCR*** 1		
Ycnc	5′-(Eclipse)-AACGCNT[A]CA-(FAM)-3′	271–280
Hcnc	5′-(Eclipse)-AACGCNC[A]CA-(FAM)-3′	271–280
Ycrc_v2	5′-(Eclipse)-AACGCRT[A]CA-(FAM)-3′	271–280
Hcrc_v2	5′-(Eclipse)-ACGCRC[A]CA-(FAM)-3′	272–280
***Synthesized NS5A-Oligonucleosides*** 2		
SYN-NS5A_Ycac	AACGCATACAC	271–281
SYN-NS5A_Hcac	AACGCACACAC	271–281
SYN-NS5A_Ycgc	AACGCGTACAC	271–281
SYN-NS5A_Hcgc	AACGCGCACAC	271–281

1 Eclipse and FAM mean quenching and fluorescent molecules, respectively. Nucleotides inside brackets indicate those replaced for RNA.

2 Each oligonucleoside was designed to target those from nt208 to nt394, but the specific sequences corresponding to those from nt271 to nt281 were shown.

Real-time PCR was carried out using the Cycleave^®^RCR Core Kit (TaKaRa Bio Inc, Seta, Japan) using the set of primers, either of the 2 cycling probe mixtures, and the cDNA derived from the RNA samples. Ycnc and Hcnc were used for amplification of cDNA from the Y93 wild-type (hereafter, Y93 wild) and Y93H mutant strains, respectively. The cycle conditions for the PCR were as follows; initial denaturation at 95°C for 30 seconds, followed by 45 cycles of denaturation at 95°C for 5 seconds, primer annealing at 55°C for 10 seconds, and extension and subsequent detection of fluorescence at 72°C for 25 seconds.

To obtain calibration curves, real-time PCR was similarly carried out using either of the 2 sets of oligonucleoside mixtures instead of the cDNA derived from the RNA samples: the SYN-NS5A_Ycac and SYN-NS5A_Ycgc mixture for quantification of cDNA from the Y93 wild strains, and the SYN-NS5A_Hcac and SYN-NS5A_Hcgc mixture for that of cDNA from the Y93H mutant strains. Each oligonucleoside was adjusted for a 100-fold dilution series from 1×10^2^ to 1×10^8^ copies/mL. The threshold cycle (*C_T_*) values were configured as the PCR products after 35 cycles for 1×10^2^ copies, which was identical to those after 10 cycles for 1×10^8^ copies in each assay. The copy percentages of the Y93H mutant strain relative to the total HCV strains were calculated based on the fluorescence levels of the PCR products for both strains.

### Direct Sequencing of the NS5A Region of HCV-RNA

A fragment with a length of 379 bases (nt16–394) corresponding to the NS5A region of HCV was amplified using 2 primer sets, namely, the external Y93_3L (nt16–33) and Y93_1R (nt376–394) primers and internal Y93_2L (nt48–66) and Y93_2R (nt346–364) primers (***Table 2***), and Tks Gflex^®^ DNA Polymerase (TaKaRa, Shiga, Japan) with primer annealing at 55°C for 10 seconds, and extension at 68°C for 15 seconds over 35 cycles in the 1st PCR and 2nd PCR. The fragment were purified using the QIAquick PCR Purification Kit (Qiagen K.K.) and sequenced using the BigDye^®^ Teminator v3.1 Cycle Sequence Kit (Applied Biosystems, CA, USA) using the internal primers, according to the protocol of the manufacturer. The nucleotide sequences of the amplified products were directly sequenced with a 3130 Genetic Analyzer (Applied Biosystems), and the obtained data for nucleotide sequences were assembled using ATGC ver.7 (GENETYX, Tokyo, Japan).

## Results

### Establishment of a Real-Time PCR System Using Cycling Probes to Quantify the RNA Levels of HCV with and without the NS5A Y93H Mutation

**Figure 1:**
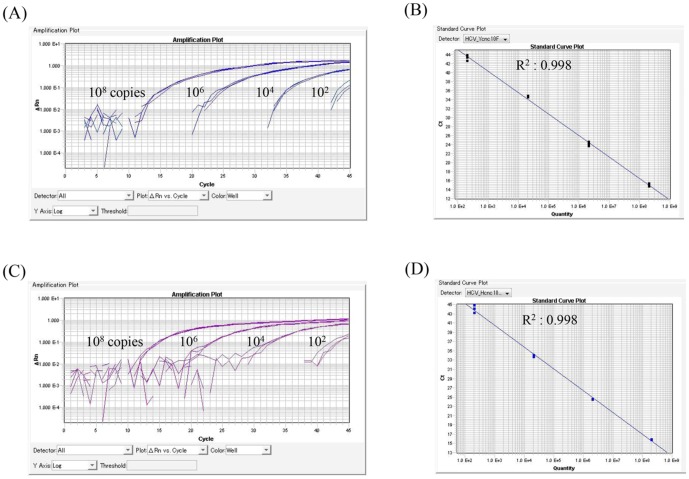
Real-Time PCR Analysis Using a Synthesized Oligonucleoside Following Adjustment for a 100-fold Dilution Series from 1×10^2^ to 1×10^8^ copies/mL. (A) Amplification plots using SYN-NS5A_Ycac. (B) The standard curve for SYN-NS5A_Ycac generated from the amplification plots displayed in Fig 1A. The correlation coefficient was 0.998. (C)Amplification plots using SYN-NS5A_Hcac. (D) The standard curve for SYN-NS5A_Hcac. The correlation coefficient was 0.998. All figures were generated by the ABI Psism 7900 Sequence Detection Software (SDS), version 2.4.

First of all, real-time PCR was carried out with the synthesized oligonucleotides following adjustment for a 100-fold dilution series. As shown in ***Figure 1***, the PCR products of SYN-NS5A_Ycac and SYN-NS5A_Hcac were quantified depending on their copy levels with Ycnc and Hcnc, respectively, as cycling probes (from 1×10^2^ to 1×10^8^ copies/mL), while no products were detected when the cycling probes were used in reverse, that is, Hcnc and Ycnc for SYN-NS5A_Ycac and SYN-NS5A_Hcac, respectively. Similar results were also obtained in the experiments using SYN-NS5A_Ycgc and SYN-NS5A_Hcgc as the synthesized oligonucleotides.

**Figure 2:**
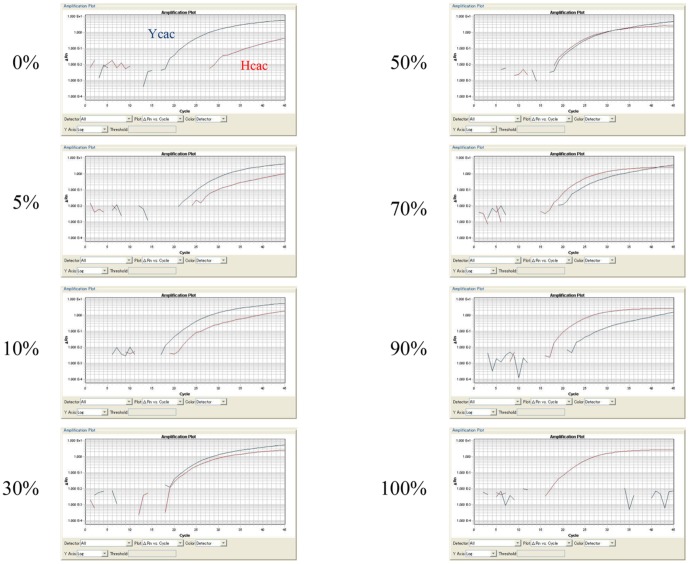
Real-Time PCR Analysis Using Mixtures of Synthesized Oligonucleoside Following Adjustment to Different Percent Volumes of Both Oligonucleosides. SYN-NS5A_Hcac/SYN-NS5A_Ycac and SYN-NS5A_Hcgc/SYN-NS5A_Ycgc were used as the mixtures and the percent volumes of both oligonucleosides were 0%, 5%, 10%, 30%, 50%, 70%, 90% or 100%. Ycac/Hcac or Ycgc/Hcgc was used as the cycling probe. The numbers on the left indicate the percent volumes of the SYN-NS5A_Hcac/SYN-NS5A_ Hcgc in each sample.

Next, real-time PCR was performed with mixtures of the synthesized oligonucleosides, SYN-NS5A_Ycac and SYN-NS5A_Hcac or SYN-NS5A_Ycgc and SYN-NS5A_Hcgc, adjusted to different percent volumes of both oligonucleosides: 0%, 5%, 10%, 30%, 50%, 70%, 90% and 100%. The results for the SYN-NS5A_Ycac/SYN-NS5A_Hcac mixtures after real-time PCR with Ycac and Hcac as the cycling probes are shown in ***Figure 2***. The PCR products of SYN-NS5A_Hcac and SYN-NS5A_Ycac were separately quantified by real-time PCR in each mixture with different proportions of both oligonucleosides; the error range in each mixture was less than 10% (***Table 3***). Similar results were obtained in experiments using SYN-NS5A_Hcgc and SYN-NS5A_Yagc as the oligonucleosides and Hcgc and Ycgc as the cycling probes (***Table 3***).

**Table 3:** The Percentages of Y93 Wild and Y93H Mutant Oligonucleosides Measured by Real-Time PCR Using Cycling Probe Mixtures Following Adjustment to Different Percent Volumes of Both Oligonucleosides.

Sample	Copies of oligonucleosides evaluated by the real-time PCR					Predicted percentages (%)
	Y93 wild		Y93H mutant		Calculated percentages of Y93H mutant (%)	
	Copies	Log	Copies	Log		
A	1.13×10^6^	6.05	0	0	0.0	0
B	1.80×10^5^	5.26	4.16×10^3^	3.64	2.2	5
C	1.05×10^6^	6.02	4.35×10^4^	4.64	4.0	10
D	1.02×10^6^	6.01	3.31×10^5^	5.52	24.4	30
E	7.89×10^5^	5.90	6.16×10^5^	5.79	43.7	50
F	3.63×10^5^	5.56	9.12×10^5^	5.96	71.5	70
G	3.39×10^4^	4.53	1.26×10^6^	6.10	97.4	90
H	0	0	9.12×10^5^	5.96	100.0	100

### Percent HCV-RNA Levels of the NS5A Y93H Mutant HCV Strains Relative to the Total HCV-RNA Levels in the Serum in Patients with HCV Infection

**Figure 3:**
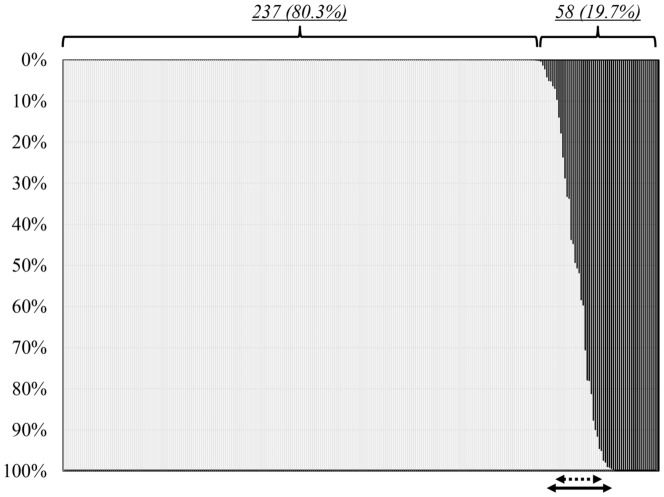
Percent RNA Levels of Y93H Mutant HCV Strains relative to the Total HCV-RNA levels in Patients with Genotype 1b HCV Infection. White and black bars indicate the percentages of Y93 wild strains and Y93H mutant strains, respectively. Y93H mutant HCV strains were undetectable in 257 patients (80.3%), while were detected in 58 patients (19.7%) with relative percentage to total HCV strains from 1.4% to 100% by Real-time PCR analysis using cycling probe mixtures Bars within the arrow and dotted arrow indicate mixed HCV strains based on real-time PCR analysis (n = 35) and direct-sequencing (n = 25), respectively.

Using the sera obtained from 332 patients with genotype 1b HCV infection, NS5A Y93 wild HCV strains and/or Y93H mutant HCV strains were quantified in 295 patients (88.9%) by real-time PCR with Ycnc and Hcnc as the cycling probe mixtures and SYN-NS5A_Ycac and SYN-NS5A_Hcac as the oligonucleoside for the calibration. The percent HCV-RNA level of the Y93H mutant HCV strains relative to the total HCV-RNA level was 0% in 237 patients (80.3%), between 1% and 99% in 35 patients (11.9%), and 100% in 23 patients (7.8%) (***Figure 3***).

The corresponding NS5A region was also amplified in the 332 patients, and direct sequencing could be carried out in 303 patients (91.3%). Among these patients, exclusively Y93 wild HCV strains were detected in 251 patients (82.8%); in which 218 patients, the percent HCV-RNA of the Y93H mutant HCV strains relative to the total HCV-RNA level was calculated as 0% by real-time PCR with the cycling probes, were included. On the other hand, exclusively Y93H mutant strains were detected in 16 patients (5.3%) by the direct sequencing method, and in these patients, 11 patients, the percent HCV-RNA of the Y93H mutant strains relative to the total HCV-RNA level was calculated as 100% by real-time PCR using the cycling probes were included. Direct sequencing revealed both Y93 wild and Y93H mutant HCV strains in 34 patients (11.2%); this percentage of patients showing quasispecies was smaller than that determined by the real-time PCR method. Among the 35 patients who showed quasispecies in respect of aa93 of the NS5A region by the real-time PCR method, no Y93H mutant strains were detected by direct sequencing in 5 patients (14.3%) in all of whom the percent HCV-RNA of the Y93H mutant strains was 14% or lower. Similarly, Y93 wild strains were undetectable by direct sequencing in 5 patients (14.3%) in whom the percentage was 88% or more (***Figure 3***). Also, Y93C mutant strains were detected in 2 patients (1.0%) by direct sequencing despite that the real-time PCR methods failed to detect such rare mutant strains.

## Discussion

**Figure 4:**
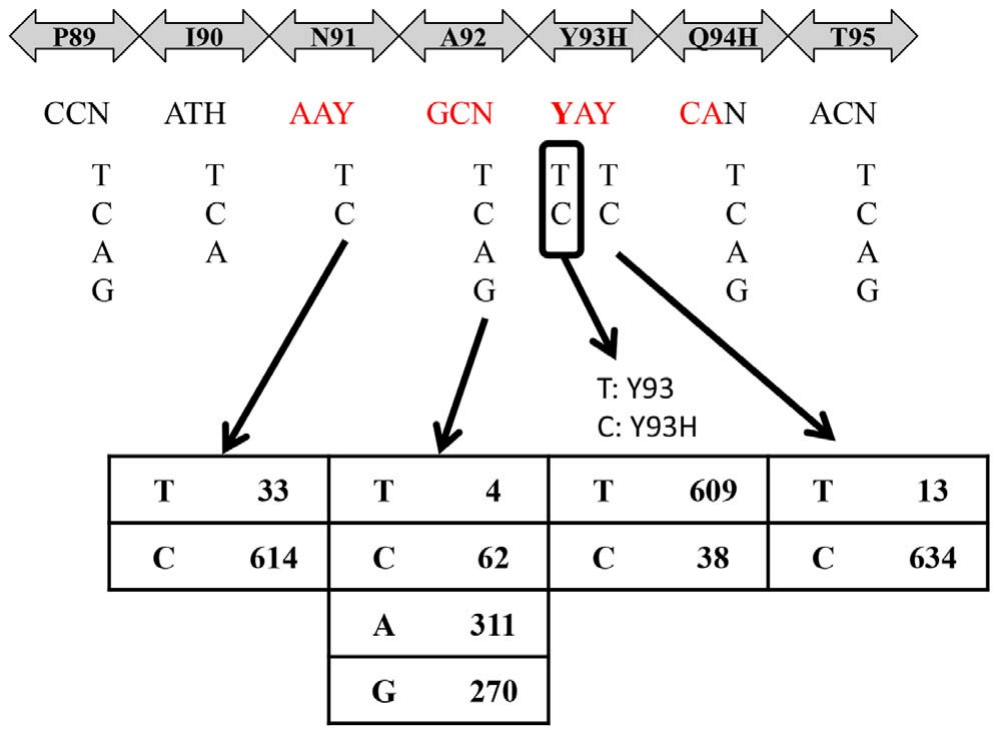
Nucleotide (nt265–285) and Amino Acid (aa89–95) Sequences in the NS5A Region of 647 Genotype 1b HCV Strains in the Database. The figures in the lower panel denote the number of HCV strains showing each nucleotide (nt273, 276, 277, 279) among the 647 genotype 1b HCV strains.

In this study, we established a real-time PCR method using cycling probes to detect and quantify HCV stains showing Y93H mutation in the NS5A region. The percent RNA of Y93H HCV strains relative to the total HCV-RNA level in the serum can be calculated by this method. In this system, both the set of primers and the 2 types of cycling probe mixtures were designed based on nucleotide sequences of genotype 1b HCV strains reported in the database [21], since dual oral therapy with daclatasvir and asunaprevir, which is expected to be approved in the near feature in Japan, has been shown to be effective exclusively in patients with genotype 1b HCV infection [18]–[19]. As shown in ***Figure 4***, a number of nucleotide polymorphisms (N at nt267, nt276, nt282, nt285, H at nt270 and T at nt283, nt279), which do not alter the amino acid sequences exist around the target nucleotide Y at nt277 in the NS5A region of HCV. Also, a nucleotide polymorphism that provokes an amino acid mutation, but with no alteration of the susceptibility to NS5A inhibitors, exists at nt282 showing N. Such abundant polymorphisms prevent detection of the Y93 wild and Y93H mutant strains by the usual PCR procedure; estimated maximal detection rates after such procedure were about 42% (614×311×609×634/647^4^ = 0.42). However, we overcame this problem through adoption of cycling probe mixtures (Ycnc and Hcnc), in which a nucleoside corresponding to nt278 was substituted for a nucleotide of the RNA. In general, cycling probes are designed to hybridize the target portion (nt277) at the RNA portion. In our preliminary experiments, however, amplification failure occurred frequently when the real-time PCR was done using such ordinary probes. The devise to shift the RNA portion enabled us to detect and quantify Y93 wild strains and Y93H mutant HCV strains separately in almost all patients with genotype 1b HCV infection. Considering that amplification failure occurred in 29 of the 332 patients (8.7%) even in the analysis by direct sequencing, in which a set of primers similar to that in the real-time PCR analysis is used, the efficacy rate of the cycling probe mixtures (Ycnc and Hcnc) for the detection of genotype 1b HCV strains was calculated as 92.5% (273/295).

It is noteworthy that our real-time PCR system established using cycling probe mixtures was more sensitive to detect Y93 wild and Y93H mutant strains separately than analysis by direct sequencing. Y93H mutant HCV strains were detected in 58 out of 295 patients (19.7%) through the real-time PCR system, while they were detected in only 50 of 303 patients (16.5%) by direct sequencing analysis. Direct sequencing failed to detect Y93H mutant HCV strains in 5 patients with serum Y93H mutant RNA/total HCV RNA ratios of 14% or less as quantified by the real-time PCR system. On the contrary, Y93 wild HCV strains were not detected by direct sequencing in 5 patients with ratios of 88% or more. In the present study, we applied our system to sera in which the ratios had been evaluated by deep-sequencing analysis (kindly provided by Prof. Nobuyuki Enomoto under approval of the institutional review boards of University of Yamanashi Hospital and Saitama Medical University) [22], and found that Y93H mutant HCV strains could be detected in sera with a percentage of 1% or more.

The limitations of our real-time PCR system were as follows. Firstly, the first set of primers used (Y93_1L and Y93_1R) was not effective to amplify the cDNA in about 9% of patients with genotype 1b HCV infection, although both primers were set targeting oligonucleotides (nt208–226, nt376–394) in the NS5A region, which shows relatively highly conserved sequences among various genotype 1b HCV strains. For such patients, a long-distance PCR using a set of primers targeting more highly conserved sequences, such as those in the core regions, was required. Secondly, amplification failure occurred in about 10% of the patients through the system using the present cycling probe mixtures (Ycnc and Hcnc). For such patients, we developed 8 different cycling probes, and the Y93 wild and Y93H mutant HCV strains could be separately quantified through the second-step real-time PCR using 2 of these 8 cycling probes selected based on information obtained by direct sequencing. Finally, a slight elevation of a background PCR noise was inevitable in the experiments using the set of primers and 2 cycling probe mixtures shown in ***Table 2***. Thus, a laboratory worker is required to check the background noises to quantify the RNA copies of the Y93 wild and Y93H mutant HCV strains. To overcome such background noises, we recently developed another set of primers and cycling probe mixtures for the first-step real-time PCR (***Table 2***). The percent RNA levels in the serum of Y93H mutant HCV strainsrelative to the total HCV-RNA can be calculated automatically by the Thermal Cycler Dice^®^ Real Time System *II* (TaKaRa Bio Inc) with no check by laboratory workers.

In conclusion, a novel system, based on real-time PCR using cycling probe mixtures, to detect and quantify Y93 wild HCV strains and Y93H mutant HCV strains separately was established. In this system, the percent RNA levels in the serum of Y93H mutant HCV strains relative to the total HCV-RNA levels could be calculated in about 90% of patients with genotype 1b HCV infection through the first-step real-time PCR. For the remaining patients, the second-step real-time PCR using another set of primers and/or 2 of 8 different cycling probe mixtures accomplished the goal. This simple system may be useful to select suitable candidates for antiviral therapy with daclatasvir and asunaprevir in Japan where genotype 1b HCV strains predominant. Also, the useful of the system for patients with genotype 1a strains is required to be investigated in the future, since daclatasvir might be combined with a higher barrier to resistance DAAs such as sofosbuvir in the USA.
